# Identification of the heart as the critical site of adenosine mediated embryo protection

**DOI:** 10.1186/1471-213X-10-57

**Published:** 2010-05-28

**Authors:** Christopher C Wendler, Ryan R Poulsen, Satish Ghatpande, Robert W Greene, Scott A Rivkees

**Affiliations:** 1 Section of Developmental Endocrinology and Biology, Yale Child Health Research Center, Department of Pediatrics, Yale University School of Medicine, New Haven, CT 06520 USA; 2 Department of Psychiatry, University of Texas Southwestern Medical Center and Dallas Veterans Administration Medical Center, Dallas, Texas 75216 USA

## Abstract

**Background:**

Our understanding of the mechanisms that protect the developing embryo from intrauterine stress is limited. Recently, adenosine has been demonstrated to play a critical role in protecting the embryo against hypoxia via adenosine A1 receptors (A1ARs), which are expressed in the heart, nervous system, and other sites during development. However, the sites of A1AR action that mediate embryo protection are not known. To determine if the heart is a key site of adenosine-mediated embryo protection, A1ARs were selectively deleted in the embryonic heart using a Cre-LoxP system in which the alpha-myosin heavy chain promoter drives Cre-recombinase expression and excision of the A1AR gene from cardiomyocytes.

**Results:**

With increasing exposure of maternal hypoxia (10% O_2_) from 48-96 hours beginning at embryonic day (E) 8.5, embryo viability decreased in the cardiac-A1AR deleted embryos. 48 hours of hypoxia reduced embryonic viability by 49% in embryos exposed from E10.5-12.5 but no effect on viability was observed in younger embryos exposed to hypoxia from E8.5-10.5. After 72 hours of hypoxia, 57.8% of the cardiac-A1AR deleted embryos were either dead or re-absorbed compared to 13.7% of control littermates and after 96 hours 81.6% of cardiac-A1AR deleted embryos were dead or re-absorbed. After 72 hours of hypoxia, cardiac size was reduced significantly more in the cardiac-A1AR deleted hearts compared to controls. Gene expression analysis revealed clusters of genes that are regulated by both hypoxia and A1AR expression.

**Conclusions:**

These data identify the embryonic heart as the critical site where adenosine acts to protect the embryo against hypoxia. As such these studies identify a previously unrecognized mechanism of embryo protection.

## Background

Hypoxia is a common fetal stressor that can be caused by placental insufficiency, maternal smoking, anemia, cord compression, preeclampsia, and living at high altitude [1]. Increasing evidence indicates that hypoxia can have profound effects on embryogenesis and influence cardiac development [2,3]. Hypoxic exposure of chicken embryos for the first 3.5 days (an equivalent of embryonic day (E) 10.5 in mouse), leads to reduced embryonic viability, growth inhibition, and altered cardiac function [4]. Long exposure to hypoxia later in development also effects cardiac development, heart function and leads to dilated cardiomyopathy [5]. Fetal hypoxia in mammals results in intrauterine growth retardation and reduced birth weight [1,6]. We also observe that hypoxia disproportionately reduces cardiac growth in embryos [3].

Evidence indicates that adenosine plays important role mediating effects of hypoxia during development [2,3,7]. Adenosine levels are dynamically regulated and increase markedly in hypoxia [8]. Adenosine acts through specific G protein-coupled receptors, including A1 adenosine receptors (A1ARs), which are activated by slight increases in adenosine levels [9,10]. A1ARs are expressed in cardiac tissue very early in embryogenesis and can regulate fetal heart function [11,12]. A1ARs are expressed in the myocardium of developing hearts beginning at E8 and cardiac A1AR expression continues throughout development [12]. In adults, over-expression of A1ARs protects the heart from ischemic/reperfusion injury, and the loss of A1ARs impairs ischemic tolerance [13,14].

We previously demonstrated that A1ARs play an essential role in protecting the embryo from hypoxia [3]. Whereas A1AR-deficient embryos do not display birth defects or growth restriction under normoxic conditions [15], embryos with global deletion of A1ARs (A1AR-/-) are severely growth retarded and have reduced viability [3]. In addition, A1AR-/- embryos expressed less stabilized hypoxia-inducible factor 1α (HIF1α) protein under hypoxic conditions and have abnormal patterns of embryonic gene expression [3]. Because A1ARs were deleted from all embryonic tissues in those studies, it was not possible to localize the embryo-protective sites of A1AR action.

During embryogenesis, A1ARs are expressed in the nodal region, the heart, and the nervous system [12]. It is also likely that A1ARs are expressed in other sites that have eluded localization via methods that have been applied. After E9.0, the embryo is dependent on the fetal heart for adequate nutrient delivery [16]. Thus isolated effects on the developing heart could induce global effects on the embryo after E9.0. To test this hypothesis, we examined the role of cardiac A1ARs in mediating embryonic protection against hypoxia through the use of a mouse model in which A1ARs are deleted in the heart, using the Cre/loxP system, with Cre-recombinase expression driven by an alpha-myosin heavy chain (α-MHC) promoter [17]. We now report that the heart is the major site where A1AR acts to confer protection against intrauterine hypoxia.

## Methods

### Animals

All experiments approved by the Institutional Animal Care and Use Committee (IACUC) of Yale University. Mice lacking cardiac A1ARs were generated by homologous recombination to introduce loxP sites around the major coding exon of the A1AR gene [18]. Cre-recombinase expression produces a truncated and non-functional A1AR gene by excising the DNA sequence between loxP sites [18]. Cardiomyocyte-specific A1AR knockout mice were generated by crossing A1AR-loxP mice with αMHC-Cre mice. A1AR-loxP (floxed; A1AR^fl/fl^) mice were generated and provided by Dr. R. Greene (Southwestern University, Dallas TX) [18]. α-MHC-Cre mice were provided by Dr. M. Schnieder (Baylor Medical Center, Houston, TX). αMHC and Cre-transgenes are expressed in the developing myocardium beginning at E7.5 [17,19].

To generate mice with A1ARs deleted from cardiomyocytes, αMHC-Cre mice were bred with A1AR^fl/fl ^mice. Male αMHC-Cre mice were mated with female mice homozygous for the A1AR floxed allele (A1AR^fl/fl^). The F_2 _matings were A1AR^fl/+, αMHC-Cre ^× A1AR^fl/fl^, from which A1AR^fl/fl, αMHC-Cre ^males were mated with A1AR^fl/fl ^females to produce the embryos used in these experiments. This mating scheme produces litters with embryos expressing A1ARs in the heart and embryos without cardiac A1ARs.

Genomic DNA was isolated from tail biopsies or embryonic yolk sacs with the DNeasy Tissue Kit from Qiagen (Valencia, CA) [20]. Genotyping PCR was performed on genomic DNA to confirm animals were A1AR^fl/fl ^and determine if αMHC-Cre was present. The presence of the A1AR floxed allele was confirmed when the forward primer (GATCCGCAAGCAGCTCAACAAA) and reverse primer (AAACTCCTCTTCAGACCTAATAAC) yielded a 619 bp band. The absence of the wild type allele was assessed with the forward primer (TGTCCTCTTCCCTTCCTGCTCCATGATTACCA) and reverse primer (AGAGACAGCGAAGCGGCGGCATATCCATTT), which yielded a 2413 bp band if the wild type allele is present. The presence of αMHC-Cre was confirmed when the forward primer (CGATGCAACGAGTGATGAGG) and reverse primer (CGCATAACCAGTGAAACAGC) yielded a 300 bp band.

### Radioreceptor Assay

To verify deletion of A1AR expression, radioreceptor assays were performed, as described [12]. Radioreceptor assays were performed on hearts and brains of postnatal day (P) 7 mouse pups using the A1AR-selective ligand [^3^H]DPCPX (1-3, dipropylcyclopentyl xanthine) [12]. Because of the small sizes of the embryos, it was not possible to assess embryonic cardiac expression.

### *In utero *Hypoxia

Pregnant mice were used to examine the effects of hypoxia on embryos, as described [3,21]. Hypoxia has been reported to increase the concentration of adenosine in the circulation to 2 μM in fetal sheep [22]; however, due to the small size of the murine embryos we were unable to determine embryonic levels of adenosine. Timed matings were used to obtain the appropriate stage embryos, with embryonic (E) day 0.5 designating the day a vaginal plug was observed. Pregnant dams were placed in a Plexiglass chamber (Biospherix, Redfield, NY) equipped with an oxygen sensor that maintains ambient O_2 _levels between 9.5-10.5% by introducing 100% medical grade nitrogen into the chamber [20]. This level of oxygen was selected because pregnant dams tolerate this concentration, and it has adverse effects on embryogenesis [3,23]. In a previous report, we validated this protocol using Hypoxyprobe-1 [3]. These experiments demonstrated that the partial pressure of oxygen (pO_2_) in embryonic tissue was less than 10 mm Hg or less than 1.3% O_2 _[3]. Control animals were housed in the same room as the hypoxia chamber in room air (21% O_2_). Pregnant dams were housed in the chamber for 48-96 hours.

### Embryo Morphology Analysis

At the end of hypoxia or normoxia exposure, pregnant dams were immediately euthanized with 100% CO_2_. Embryos were collected in Dulbecco's phosphate buffered saline (D-PBS, Invitrogen, Carlsbad, CA) and assessed for viability. Embryos with beating hearts were scored as alive; embryos without beating hearts were scored as dead; when no embryo was collected from a uterine bud, it was scored as reabsorbed. The viability rate was calculated as the number of live embryos divided by the total number of embryos collected, including dead and reabsorbed.

Embryonic and cardiac morphology was assessed using a Zeiss Stemi 2000-C dissecting microscope (Carl Zeiss, Oberkochen, Germany). Digital images of whole embryos were captured with an Olympus C-5060 camera (Olympus, Tokyo, Japan). Quantitative measurements of the crown-rump (CR) length were taken from photographic prints.

Embryos were fixed, embedded in paraffin, and sectioned, as described [24]. Sections were stained with Harris-modified hematoxylin and Eosin Y (H&E, Fisher Scientific, Fair Lawn, NJ), according to standard procedures. Images were captured under bright field conditions with an Olympus IX70 inverted microscope and analyzed for cross sectional area of various cardiac structures using Image-Pro Plus analysis software (Media Cybernetics, Inc., Silver Spring, MD). Area analysis was taken from sagittal sections of embryos at the level of the junction of the atrioventricular (AV) canal and the ventricle. The area of the ventricular myocardium, AV canal cushions or outflow tract (OFT) cushions were outlined and the area determined by imaging software. Every third section was analyzed, and 5-6 sections per embryo were quantitated. The average area for each cardiac structure was determined for each embryo.

### Microarray Analysis

Total RNA was purified from isolated hearts exposed to *in utero *hypoxia (10% O_2_) for 24 hours from E9.5 to E10.5, as described [3]. RNA was collected from at least 10 hearts from each group (Normox/Flox, Normox/Cre, Hypox/Flox, and Hypox/Cre). The RNA was then provided to the W. M. Keck Foundation Biotechnology Resource Laboratory at Yale University for analysis. Gene expression analysis was performed using the Illumina microarray gene chip Mouse 6 (Illumina Inc., San Diego, CA), which interrogates the whole mouse genome of over 30,000 genes. Each analysis was run in triplicate. Changes in gene expression (fold change) and p-values were calculated using Partek software (St. Louis, MO) at the Yale Center for Statistical Genomics and Proteomics. Analysis of gene pathways was performed using Metacore software (Genego Pathway Analysis Inc., San Diego, CA).

### Statistical Analysis

Data are presented as means +/- the standard error of the mean (SEM). Analyses were performed with the statistics software package included with Microsoft Excel (Microsoft, Edmond, WA) and GraphPad Prism (GraphPad Software Inc., San Diego, CA). Statistical comparisons between groups were performed with student *t *tests (two sample assuming equal variances), ANOVA, or the Mann-Whitney test. *P *< 0.05 was considered to be statistically significant.

## Results

### Deletion of cardiac A1ARs results in reduced embryonic viability in hypoxia

We first verified deletion of A1AR expression in the heart by radioreceptor assay [12], using the A1AR-selective ligand [^3^H]DPCPX (1-3, dipropylcyclopentyl xanthine) [12]. In the A1AR^fl/fl ^mice, the concentration of A1ARs in brain and heart were 28.2 + 4.2 and 2.2 + 1.1 fmol/mg of protein, respectively. However, in the A1AR^fl/fl, αMHC-Cre ^mice, concentrations of A1ARs in brain and heart were 29.7 + 3.0 and 0.0 + 0.0 fmol/mg of protein, respectively. Thus, there is no A1AR expression in the hearts of the A1AR^fl/fl, αMHC-Cre ^mice.

To examine the role of cardiac A1ARs in protecting the developing embryo from hypoxia, pregnant dams were exposed to hypoxia for 48, 72, or 96 hours. We first examined the viability of embryos following hypoxic exposure. Each embryo or uterine bud was scored as either alive (heart beating), dead (embryo intact but no beating heart), or reabsorbed (no recognizable tissue inside a uterine bud.

**Figure 1:**
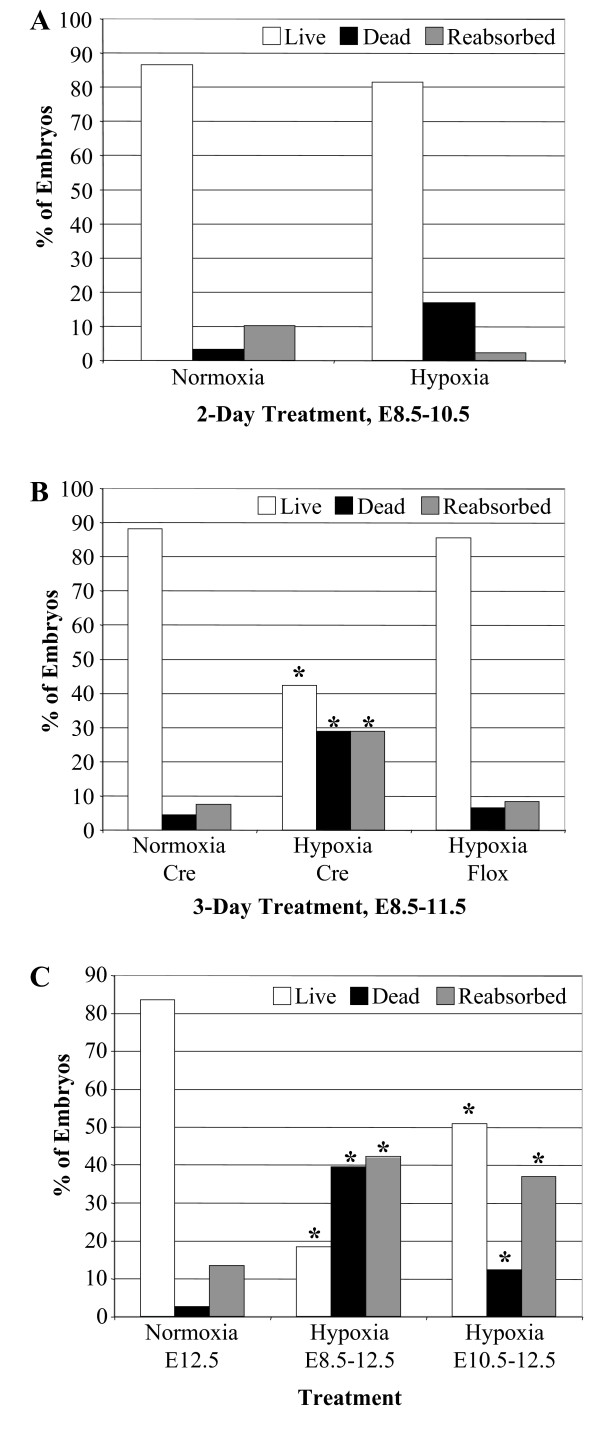
**Cardiac specific knockout of A1AR reduces embryonic viability under hypoxic conditions**. Pregnant dams were reared in room air or hypoxia (10% O_2_) for 2, 3, or 4 days beginning at E8.5. (A) 2-day treatment from E8.5-10.5, no difference in viability between normoxia and hypoxia, P > 0.05. (B) 3-day treatment from E8.5-11.5, hypoxia induced a decrease in viability of 52% in the A1AR cardiac-specific knockout mice but not in the normal A1AR expressing (Hypoxia +/+) litters. (C) 4-day treatment from E8.5-12.5, hypoxia caused a greater reduction in viability of 78% compared to normoxia controls. In addition, 2-day treatment from E10.5-12.5 induced a significant decrease in viability of 40% that was not seen in the younger 2-day treated (E8.5-10.5) embryos. *, P ≤ 0.05 by Mann-Whitney test with a 99% confidence level.

When pregnant dams were treated for 48 hours from E8.5 to E10.5, there were no significant differences between normoxia (room air) and hypoxia (10% O_2_) exposure in the percentage of live embryos (Fig. 1A). The viability (percentage of live embryos) for the 48 hours groups was 86.4% (51 alive out of 59 total embryos) for normoxia and 81.3% (39/47) for hypoxia treatment groups, respectively. These percentages are consistent with what we observed for all normoxia groups examined, and ranged from 80-90% viability.

We next examined effects of 72 hours of hypoxic exposure *in utero*, from E8.5-11.5. 88.1% (82/93) of embryos in normoxic dams were viable, and 42.2% (35/83) were viable in the hypoxia-exposed dams (Fig. 1B). This decrease in viability was reflected in an increase in the number of dead embryos and an increase in the number of reabsorbed embryos (Fig. 1B). The low level of dead embryos 4.3% (4/93) and reabsorbed embryos 7.5% (7/93) in room air increased to 28.9% (24/83) for both dead and reabsorbed embryos after 72 hours of hypoxia (Fig. 1B).

To confirm that reduced viability was due to the loss of cardiac A1ARs, A1AR^fl/fl ^females were mated with A1AR^fl/fl ^males without the Cre-recombinase gene. When we examined the litters from A1AR^fl/fl ^matings exposed to 72 hours of hypoxia, embryo viability was 85.4% (41/48) similar to that of the normoxia/Cre group (Fig. 1B). Thus, loss of viability is A1AR dependent.

We next examined the effects of 96 hours of hypoxia. Previous work examining the role of A1AR in protecting the embryo from hypoxic stress demonstrated reduced viability and increased percentage of reabsorbed embryos after 96 hours of hypoxia in the A1AR global knockout (KO) mouse model [3]. Four days of hypoxia exposure from E8.5-12.5 caused a significant decrease in embryo viability and an increase in embryo re-absorption in the cardiac specific A1AR KO mice (Fig. 1C). Compared with 72 hours, 96 hours of hypoxia had a much greater adverse effect on viability; the viability for 96 hours went from 83.8% (31/37) in room air to only 18.4% (7/38) in hypoxia (Fig. 1C).

To further define periods of vulnerability, we examined hypoxia from E10.5-12.5, 48 hours of hypoxia in older embryos also reduced viability to 50.8% (33/65) and increased re-absorption of embryos to 36.9% (24/65, Fig. 1C). This finding is in contrast to the viability observed for the 48 hours of hypoxia treatment earlier in development from E8.5-10.5 (81.3%). These observations indicate that the period of E10.5-12.5 is when embryo protection against hypoxia is most dependent on cardiac A1ARs.

### Influences on cardiac development

Previous work has demonstrated that hypoxia exposure early in embryogenesis leads to growth retardation and reduced cardiac tissue growth, both of which are exacerbated by the global loss of A1ARs [3,21]. To test if deletion of cardiac A1ARs mediated effects on embryonic and cardiac growth, we assessed the CR length of embryos following 48, 72, and 96 hours of hypoxia and cardiac tissue area after 72 hours of hypoxia. CR lengths and heart size measurements were obtained from live embryos only. Four groups of embryos were analyzed: 1) Normoxia A1AR^fl/fl ^(Normox/Flox) dams reared in room air. 2) Normoxia A1AR^fl/fl, αMHC-Cre ^(Normox/Cre) dams reared in room air and A1ARs deleted in cardiomyocytes. 3) Hypoxia A1AR^fl/fl ^(Hypox/Flox) dams reared in 10% O_2_. 4) Hypoxia A1AR^fl/fl, αMHC-Cre ^(Hypox/Cre), dams reared in 10% O_2 _and A1ARs deleted in cardiomyocytes.

**Figure 2:**
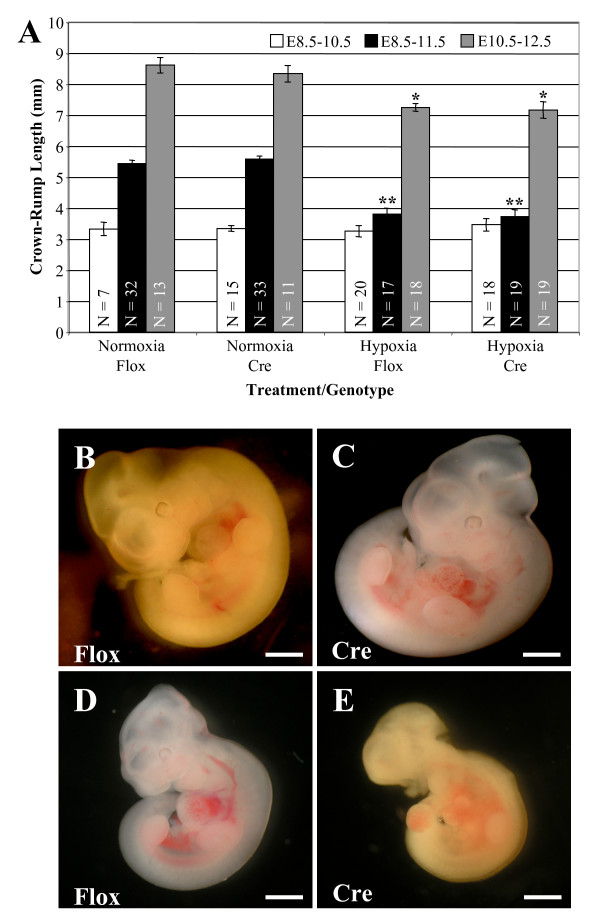
**Hypoxia exposure for three days induces embryonic growth retardation**. (A) Embryos exposed to hypoxia for 2 days *in utero *from E8.5-10.5 were not growth retarded compared to room air controls, but embryos exposed to hypoxia *in utero *from E10-12.5 exhibited significant growth retardation. Embryos exposed to hypoxia for 3 days demonstrated an even greater amount of growth retardation compared to controls. (B-E) Representative pictures of the different treatment and genotype groups after 3 days are shown. Normoxic embryos both (B) Normox/Flox and (C) Normox/Cre displayed normal morphology and growth. The hypoxic embryos (D) Hypox/Flox and (E) Hypox/Cre were significantly growth retarded, however there was no difference between Hypox/Flox and Hypox/Cre embryos. Cre/+ embryos have A1AR deleted from cardiomyocytes. *, P ≤ 0.01; **, P ≤ 0.001. Scale bar is 1 mm.

After 48 hours of hypoxia from E8.5-10.5, there was no significant difference in CR lengths compared to normoxia controls. There are no differences among all four groups (Fig. 2). However, hypoxia for 48 hours from E10.5-12.5 resulted in significant embryo growth inhibition as compared to room air controls (Fig. 2). Both the Hypox/Cre and the Hypox/Flox groups were growth retarded by about 15% after 48 hours of hypoxia at these later stages, but there was no difference between these two hypoxia groups (p > 0.05; Fig. 2).

After 72 hours of hypoxia, from E8.5-11.5, both the cardiac A1AR KO embryos and the control littermates exhibited significant growth retardation, with CR length 30% less than room air controls (Fig. 2E). No specific gross morphological defects were identified in the 72 hours hypoxic embryos (Fig. 2A-D). Although both Cre and non-Cre embryos were affected by hypoxia, there were no differences in crown-rump length between the Hypox/Cre and Hypox/Flox groups after 72 hours of hypoxia (Fig. 2). This observation may be due to the fact that only live embryos were measured and some of the more severely affected embryos were not counted. This issue would affect the Cre group more, as they have a higher rate of dead and re-absorbed embryos.

When we examined heart morphology, we observed retarded heart growth in embryos lacking cardiac A1ARs under hypoxia, compared to embryos with cardiac A1ARs. Embryos exposed to hypoxia for 72 hours from E8.5-11.5 were studied as described [3]. 72 hours of exposure was chosen for this analysis, as no discernable effects on embryonic growth were seen with 48 hours (from E8.5-10.5), and 96 hours of hypoxia exposure led to greatly decreased viability.

We measured three areas of hearts to determine the effects of hypoxia and loss of cardiac expression of A1AR on the growth of the developing heart. We calculated the average area of the myocardium throughout the heart and in the AV canal region of the heart. In addition, the areas of both the AV canal and outflow tract (OFT) cardiac cushions were measured.

**Figure 3:**
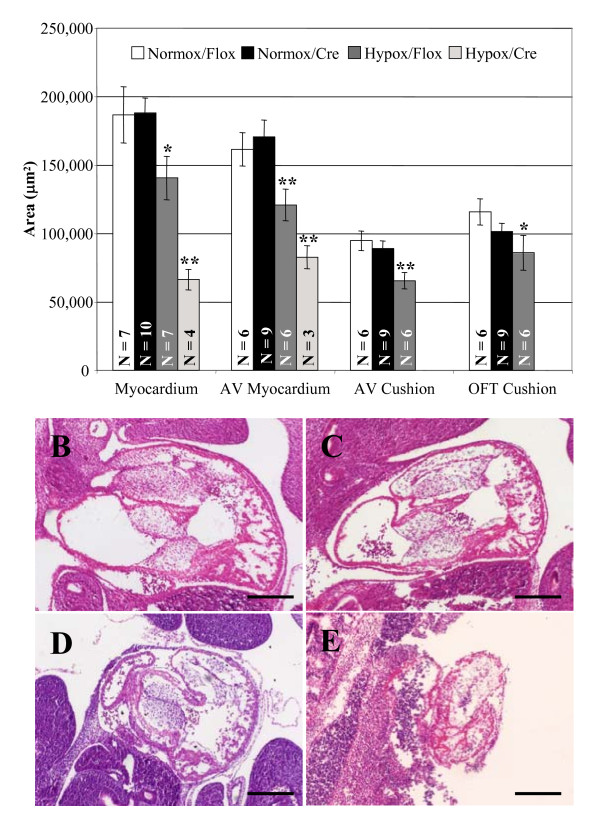
**Loss of A1ARs in cardiac tissue leads to reduced heart tissue under hypoxic conditions**. Pregnant dams were exposed to hypoxia for 3 days from E8.5-E11.5 before embryos were collected. (A) Using Image-Pro Plus software, the cross-sectional area of the ventricle throughout the heart and at the level of the AV canal was measured as well as the area of the AV and OFT cardiac cushions. Hypoxia caused a 25% decrease in the area of myocardial tissue both overall and at the level of the AV canal in the Hypox/Flox group. In the Hypox/Cre group, with A1AR deleted from cardiomyocytes, the decrease in area was more severe, 65% overall and 52% at the level of the AV canals. The AV canal cushions (30%) and OFT cushions (26%) were reduced in size in the Hypox/Flox group. Both room air groups (B) Normox/Flox and (C) Normox/Cre exhibited normal morphology and size. (D) Hypox/Flox hearts were normal in morphology but truncated in size, where as the (E) Hypox/Cre hearts displayed disrupted morphology and severely reduced size. *, P ≤ 0.05; **, P ≤ 0.01 by student's t-test. Scale bar is 200 μm.

Our analysis demonstrated a decrease in cardiac myocardium by 25% for both total and AV canal region myocardial tissue in the Hypoxia/Flox group compared to the control Normox/Flox group (Fig. 3). The Hypox/Cre group showed an even greater reduction in size of 65% for total and 52% for AV region myocardium compared to its control group Normox/Cre (Fig 3). Histological analysis confirmed the marked decrease in size of the Hypox/Cre hearts and the disrupted morphology compared to the Hypox/Flox hearts, which demonstrate reduced size but display normal overall morphology similar to room air controls (Fig. 3A-D). The cardiac cushions, both AV and OFT, were also analyzed, and the Hypox/Flox group had a similar, 30% and 26%, respectively, decrease in area as seen for myocardial analysis tissue (Fig. 3E).

Because of decreased viability of the Hypox/Cre group, fewer hearts were analyzed by this method, and there was not enough tissue to accurately measure the AV canal and OFT cushions. These data indicate that although the overall growth inhibition of the whole embryo observed with hypoxia exposure is not exacerbated by the loss of cardiac A1AR expression, the growth and morphology of the heart is adversely affected by hypoxia to a greater degree when A1ARs were deleted from cardiomyocytes.

96 hours of hypoxia caused an even greater reduction in growth but few embryos were collected and measured. The average crown rump length of Hypox/Flox embryos exposed to hypoxia for 96 hours, from E8.5-12.5, was decreased by 34% to 5.7 mm (± 0.29, N = 5), which is extremely significant compared to 8.6 mm for Normox/Flox embryos (P ≤ 3.7 × 10^-6^). There were not enough 96 hours Hypox/Cre embryos to analyze for crown-rump length. These data indicate that hypoxia inhibits overall embryonic growth but the loss of cardiac A1AR expression does not exacerbate hypoxic growth inhibition.

### A1AR-mediated gene expression

Microarray analysis was performed on RNA collected from hearts following 24 hours of hypoxia exposure to identify molecular pathways involved in A1AR-mediated embryonic protection. Genes with altered expression in A1AR-deleted hearts were involved in biological pathways important for hypoxia responsiveness and normal heart development (Table 1).

**Table 1:** Pathways affect by Cardiac A1AR loss.

Cellular Process/Pathway	# of Genes	P Value
Transcription/Role of Akt in hypoxia induced HIF1 activation	7 of 26	4.75E-04
Cytoskeleton remodeling/Regulation of actin cytoskeleton by Rho GTPases	6 of 23	1.47E-03
Glycolysis and gluconeogenesis (short map)	8 of 37	9.28E-04
Development/EPO-induced PI3K/AKT pathway and Ca(2+) influx	7 of 32	1.81E-03
Cytoskeleton remodeling Role of PKA in cytoskeleton reorganization	6 of 31	7.28E-03

Two sets of genes were identified to have altered expression with the loss of A1AR gene expression: In the first set, gene expression was influenced by hypoxia when A1ARs were not expressed in the cardiomyocytes (Table 2). The second set of genes had altered expression with the loss of A1AR expression either under hypoxic or normoxic conditions (Table 3). Genes with altered expression include, myosin heavy chain alpha (Myh6) and myosin heavy chain beta (Myh7), both of which are important for cardiac development and they are expressed throughout the heart early in development but become more restricted as development continues [25]. The expression of these myosin genes was disrupted by the loss of cardiac A1ARs. Myh6, which normally decreases at E10.5, was up regulated in hearts without A1ARs (Table 3). In comparison, Myh7 was down regulated in A1AR deleted hearts compared to hearts with A1AR expression (Table 3).

**Table 2:** Genes Regulated by Hypoxia and A1AR.

Probe ID	Gene	Hypox/Flox vs. Normox/Flox	P-Values	Hypox/Cre vs. Normox/Cre	P-Values	Hypoxia, A1AR, or Cardiac Related
ILMN_2675874	Alas2	1.91597	2.06E-06	-3.18773	2.30E-08	Hypoxia inducible
ILMN_1219154	Mt2	1.63401	0.000359711	-2.98376	1.07E-06	Hypoxia
ILMN_2596522	Mt1	2.56154	0.000118805	-2.50707	0.000139519	Hypoxia
ILMN_1250195	Ndrg1	1.9098	5.38E-06	-2.29342	8.07E-07	Hypoxia inducible
ILMN_2998934	Cited4	1.69681	8.45E-06	-2.17613	4.47E-07	Hypoxia
ILMN_2606746	Car2	1.5989	0.00168738	-2.14858	6.64E-05	Hypoxia
ILMN_2881620	Nfe2	1.87921	1.62E-06	-1.93972	1.11E-06	Cardiac
ILMN_2757966	Cxcl4	2.27996	1.05E-05	-1.89452	6.70E-05	Hypoxia inducible
ILMN_2652909	Ddit3	1.88154	0.00576158	-1.72809	0.0120442	Hypoxia inducible
ILMN_2829594	Hspa1a	2.36474	1.80E-05	-1.61118	0.00104107	Hypoxia, Cardiac
ILMN_2739544	Stc2	1.92291	2.06E-05	-1.5783	0.000261328	Hypoxia
ILMN_2605819	Egln3	2.16962	1.28E-05	-1.49541	0.00117156	Hypoxia inducible
ILMN_2643513	Asns	1.63692	5.53E-05	-1.47277	0.00029547	Hypoxia inducible
ILMN_2616565	Slc2a3	2.08188	0.000497509	-1.42853	0.0256759	Hypoxia inducible
ILMN_1249378	Bhlhb2	1.89041	4.28E-05	-1.41041	0.00250315	Hypoxia inducible
ILMN_1252762	Trib3	1.5549	2.61E-05	-1.14939	0.0267126	Hypoxia inducible
ILMN_2993109	Ddit4	1.53476	0.000963138	-1.14033	0.158638	Hypoxia inducible
ILMN_2747959	Dcn	-1.59119	2.13E-06	-1.02086	0.608063	Hypoxia inducible
ILMN_1234662	Mb	-1.51848	0.00354732	-1.00718	0.946075	Hypoxia inducible
ILMN_2632940	Sumo1	1.59231	1.31E-05	-1.00062	0.990303	Hypoxia, A1AR, Cardiac
ILMN_3001540	Lum	-1.64593	4.50E-05	1.15664	0.0484385	Hypoxia, Cardiac
ILMN_2639036	Hspd1	1.61697	0.000950629	1.1836	0.112501	Hypoxia, Cardiac
ILMN_2763002	Eno2	1.72159	4.71E-05	1.22827	0.0171715	Hypoxia
ILMN_2677662	Hspb7	-1.89271	6.57E-05	1.59123	0.000573211	Cardiac

**Table 3:** Genes Regulated by A1AR.

Probe ID	Symbol	Hypox/Cre vs. Hypox/Flox	P-Values	Normox/Cre vs Normox/Flox	P-Values	Hypoxia, A1AR, or Cardiac Related
ILMN_2665239	Myh7	-1.97085	0.000711557	-1.72806	0.0026561	Cardiac
ILMN_2697652	Chd4	-1.8584	1.21E-05	-1.60686	8.43E-05	Cardiac
ILMN_2610234	Il15	-1.83954	3.66E-05	-1.39673	0.00201565	Hypoxia, Cardiac
ILMN_2870672	Fbln1	-1.51609	0.000181497	-1.507	0.00020053	Cardiac
ILMN_2489448	Sobp	-1.50775	0.000618205	-1.54295	0.000432693	Hypoxia
ILMN_2655260	Ptp4a3	1.51792	0.00020671	1.95951	6.64E-06	Cardiac
ILMN_2967266	Fxyd5	1.52066	0.000483488	1.53701	0.000409621	Hypoxia
ILMN_3155180	Itpr2	1.52132	0.0152983	1.57414	0.0105084	Cardiac
ILMN_2711267	Krt18	1.63969	0.000254672	1.58581	0.000406559	Cardiac
ILMN_3139103	Adam15	1.68522	0.000507188	1.48327	0.00285996	Cardiac
ILMN_2475156	Xist	1.98732	0.00101892	2.03269	0.00083102	Hypoxia, Cardiac
ILMN_1213376	Cfl1	2.02757	8.54E-08	2.19178	3.76E-08	Cardiac
ILMN_2640008	Myh6	2.0374	0.00470426	1.59291	0.0349642	Cardiac
ILMN_2761594	Cirbp	2.39335	1.52E-07	1.79484	3.32E-06	Hypoxia, Cardiac

## Discussion

A1ARs are not critical for normal embryonic or cardiac development in normoxic conditions, as deletion of A1ARs throughout the embryo does not result in birth defects or growth retardation under normal circumstances [15]. During hypoxia however, A1ARs play an essential role in protecting the embryo [3]. Studying murine embryos lacking A1ARs in the heart, we now identify the heart as the critical region where A1ARs act to confer embryo protection.

Previous studies indicate that embryo viability was greatly decreased after 96 hours of hypoxia when A1AR was deleted globally [3]. In the current studies, we examined the viability of cardiac-deleted A1AR mice after 48, 72, and 96 hours of hypoxia exposure (10% O_2_). We found that after 48 hours of hypoxia from E8.5-10.5, there was no difference in viability between normoxia and hypoxia treated embryos. Since the heart becomes more important for nutrient delivery and embryo survival as embryogenesis proceeds, we examined whether 48 hours of exposure later in development, from E10.5-12.5, could affect embryonic viability. Two days later in development, the embryo is more susceptible to hypoxic stress, as embryos exposed to hypoxia from E10.5-12.5 had a 40% decrease in viability compared to room air controls.

After 72 hours of hypoxia, from E8.5-11.5, we observed a dramatic decrease in embryo viability compared to room air controls. We were able to determine the genotype of live embryos by testing yolk sac DNA, but we were not able to genotype all the dead and reabsorbed embryos. Since we were unable to determine that only Cre-expressing embryos were dying, we mated A1AR^fl/fl ^mice with each other. These mice did not express Cre-recombinase, therefore cardiac A1AR expression was normal. Under hypoxic conditions, the viability of non-Cre litters was the same as room air control litters, indicating that the loss in viability observed in the cardiac A1AR knockout litters was due to the loss of A1AR and not hypoxic exposure alone. Finally, we examined embryonic viability after 96 hours of hypoxia exposure, from E8.5-12.5, in this case the viability was much less than the 72-hour hypoxia group with less than 1 in 5 embryos surviving. These results are similar to that observed with the global knockout of A1AR after 96 hours of hypoxia [3]. Collectively these data indicate that older embryos are more susceptible to hypoxic insults, and cardiac A1ARs are critical in protecting embryos from hypoxic conditions.

Next, we analyzed embryonic growth of all live embryos collected by measuring the crown-rump length. After 48 hours of treatment between E8.5-10.5, there were no differences in CR lengths, the same result as that seen with embryo viability at this stage. However, hypoxic exposure for 48 hours later in development caused a small but significant decrease in embryo size compared to room air controls, but no difference between the hypoxia groups with and without cardiac A1AR expression was observed. Growth inhibition was also analyzed after 72 hours of hypoxia, from E8.5-11.5, embryos were more severely growth retarded than the later 48 hours of hypoxia exposure but again no differences between the hypoxia exposed groups were observed. Due to the poor viability of 96 hours hypoxia exposed embryos, we could not get an accurate measure of the crown-rump length. These data are consistent with results for non-transgenic C57Bl/6 mice exposed to hypoxia for 72 hours, which showed a 24% growth inhibition [3].

Although cardiac A1ARs are critical for viability during hypoxic stress, their loss does not increase the amount of growth inhibition seen with hypoxia treatment. The growth inhibition observed in the cardiac A1AR knockout mice is consistent with that in wildtype mice (C57Bl/6 strain) exposed to hypoxia [3]. We previously reported that global loss of A1ARs affects overall embryonic growth above and beyond what is observed in non-transgenic mice exposed to hypoxia for 96 hours [3]. However, that previous report only classified embryos into two categories, collected embryos and re-absorbed embryos, and did not make a determination of whether the collected embryos were alive or dead. This current report classifies embryos into three categories, live, dead, and re-absorbed. So when embryonic growth, as measured by crown-rump length, is compared only between live embryos of the different genotypes, the loss of cardiac A1AR does not exacerbate hypoxia growth inhibition. These data indicate that hypoxia growth inhibition is a general effect of hypoxia and that reduced viability in hypoxia is specific for the loss of A1AR in the heart.

The loss of cardiac A1ARs has severe adverse effects on heart development. 72 hours of hypoxia exposure from E8.5-11.5 causes a 65% decrease in myocardial tissue in hearts not expressing A1ARs compared to only a 25% decrease in cardiac tissue in hearts expressing A1ARs. There was also a decrease in the size of the cardiac cushions following hypoxia in hearts expressing A1ARs, however hearts without A1AR expression were too small and malformed to measure the cardiac cushion tissue accurately. These data indicate that the loss of A1ARs in cardiomyocytes is having severe adverse effects on cardiac development when the embryo is exposed to hypoxic conditions. Reduced cardiac tissue could be leading to abnormal function of the embryonic heart and that maybe why there is reduced viability at the later stages of development when cardiac function becomes critical for embryonic survival.

Because of the small sizes of the embryos studied, it was not possible for us to directly assess levels of the other adenosine receptors in the myocardium. However, during early embryogenesis, only A1ARs are known to be expressed in the heart [12]. In studies of global A1AR-/- mice, changes in the expression of other adenosine receptors have not been observed [15]. Due to technical limitations, it was also not possible for us to assess adenosine levels in the normoxic or hypoxic embryos.

Gene expression analysis revealed sets of genes in the heart that are misregulated during hypoxia exposure when A1ARs are not expressed. For example, there are genes that are misregulated with the loss of A1AR expression either in hypoxic or normoxic conditions, such as Myh6 and Myh7. Gene expression data thus indicates that A1AR loss could adversely affect cardiac development by altering the expression of cardiac genes related to cardiac function, leaving the embryo susceptible to hypoxic stress.

Further analysis reveals another set of genes that are miss-regulated in the A1AR deleted hearts. These genes are normally up regulated in response to hypoxia to protect the heart from damage including heat shock proteins 60 (Hspd1) and 70 (Hspa1a), and Metallothionein (MT) 1 and 2, but in the cardiac A1AR-/- hearts their expressions are down regulated or not changed with hypoxia treatment [26-29]. A1AR signaling was demonstrated to up-regulate MT levels during ischemia-reperfusion (I/R) and may mediate the cardioprotective effects of A1AR agonist preconditioning [27]. MT has been reported to stabilize HIF protein and this may be a mechanism of cardio protection [30], and relates to the fact that we demonstrated less stabilized HIF protein during hypoxia in the global knockout A1AR embryos [3].

These data indicates that the mechanism by which A1AR mediates cardio and embryonic protection during hypoxic exposure of embryos *in utero *may be similar to the mechanisms by which A1AR signaling leads to cardioprotection from I/R injury in adult hearts and may be related to HIF signaling. Gene expression analysis also indicates that the loss of A1AR signaling may leave the heart more susceptible to hypoxia-induced damage since increased expression of the genes associated with protection against hypoxia were not observed.

## Conclusions

Our findings confirm that A1AR expression is important for embryonic survival during hypoxic stress and that the A1ARs expressed in the heart mediate this protection. Disrupted cardiac development during hypoxia exposure in cardiac A1AR deleted embryos leads to decreased embryonic viability at stages where cardiac function is critical for embryonic survival.

## Abbreviations

(A1ARs): Adenosine A1 receptors; (E): embryonic day; (αMHC): alpha-myosin heavy chain; (1-3, dipropylcyclopentyl xanthine): DPCPX; (CR): crown-rump; (AV): atrioventricular; (OFT): outflow tract; (Myh6): myosin heavy chain alpha; (Myh7): myosin heavy chain beta; (MT): Metallothionein; (HIF1α): hypoxia-inducible factor 1α.

## Authors' contributions

CCW designed experiments, analyzed data and wrote the manuscript. RRP performed histology experiments and analyzed microarray data. SG performed hypoxia experiments and analyzed data. RWG provided the A1AR Flox mice. SAR designed experiments, analyzed data, and edited the manuscript. All authors have read and approve this manuscript.
